# A Novel Ultrasound-Guided Bilateral Vagal Nerve Hydrodissection With 5% Dextrose Without Local Anesthetic for Recalcitrant Chronic Multisite Pain and Autonomic Dysfunction

**DOI:** 10.7759/cureus.63609

**Published:** 2024-07-01

**Authors:** King Hei Stanley Lam, Daniel Chiung-Jui Su, Yung-Tsan Wu, Giustino Varrassi, Teinny Suryadi, K. Dean Reeves

**Affiliations:** 1 Board of Clinical Research, The Hong Kong Institute of Musculoskeletal Medicine, Kowloon, HKG; 2 Faculty of Medicine, The Chinese University of Hong Kong, New Territories, HKG; 3 Faculty of Medicine, The University of Hong Kong, Hong Kong, HKG; 4 Physical Medicine and Rehabilitation, Chi Mei Medical Center, Tainan, TWN; 5 Physical Medicine and Rehabilitation, Tri-Service General Hospital, Taipei, TWN; 6 Pain Medicine, Paolo Procacci Foundation, Rome, ITA; 7 Physical Medicine and Rehabilitation, Synergy Clinic, Jakarta, IDN; 8 Physical Medicine and Rehabilitation, Hermina Podomoro Hospital, Jakarta, IDN; 9 Rehabilitation Medicine, Private Practice, Kansas City, USA

**Keywords:** chronic multisite pain, autonomic dysfunction, 5% dextrose in sterile water, chronic pain management, carotid sheath, vagus nerve, ultrasound-guided hydrodissection

## Abstract

Chronic pain is a complex condition that often poses diagnostic and management challenges due to its multifactorial etiology. This case report describes a 49-year-old pastor who presented with a three-year history of chronic pain affecting multiple sites, including the neck, bilateral shoulders, thoracic region, lower back, and bilateral knees. Additionally, he experienced shortness of breath on mild exertion, which adversely affected his ability to converse and speak publicly. The patient had a rapid resting heart rate of 100-120 beats per minute, occasional palpitations, and a 24-hour electrocardiogram that confirmed 15% premature ventricular complexes with bigeminy and trigeminy. He complained of limited appetite with early satiety, intermittent nausea, and regurgitation. Despite consultations with multiple specialists, no underlying causes were identified in the cardiac, respiratory, gastrointestinal, or psychological domains. Ultrasound-guided bilateral vagus nerve hydrodissection using 5% dextrose without local anesthetics was administered three times at monthly intervals, resulting in remarkable pain relief within three months and the effects persisted at the nine-month follow-up. Tachycardia was no longer perceived, resting heart rate slowed to 70-80 beats per minute, shortness of breath improved, and public speaking ability was restored. The patient's early satiety, nausea, and reflux complaints were resolved. This case report highlights the potential effectiveness of this novel intervention for chronic pain. Further research is warranted to validate these findings and explore the mechanism of action.

## Introduction

Chronic pain is a complex and prevalent condition that poses diagnostic and management challenges due to its multifactorial etiology. It affects a significant portion of the population, with reported prevalence rates ranging from 10% to 55%, depending on the specific population studied [1]. The burden of chronic pain extends beyond the physical symptoms, impacting the quality of life, daily functioning, and mental well-being of affected individuals.

The vagus nerve, also known as the 10th cranial nerve, plays a critical role in the regulation of various bodily functions, including cardiac activity, respiratory function, gastrointestinal processes, and immune, endocrine, and autonomic systems. Dysfunction or altered activity of the vagus nerve has been implicated in the pathophysiology of chronic pain [2,3]. Targeting the vagus nerve through therapeutic interventions has gained attention as a potential avenue for managing chronic pain and its associated symptoms. Invasive cervical vagus nerve stimulation (VNS) is approved for the treatment of epilepsies, depression, obesity, and stroke rehabilitation [4]. The United States Food and Drug Administration also approves VNS for treating migraines and cluster headaches [5]. Invasive VNS requires surgery with its inherent side effects and is expensive and not readily available. Non-invasive transcutaneous VNS has been developed to enhance autonomic balance and function in various autonomic, neurological, psychiatric, rheumatologic, as well as other diseases but its efficacy and durability need further confirmation, and only left-sided VNS is recommended due to concerns about stimulating bradycardia [5]. 

Nerve hydrodissection with dextrose 5% in water (D5W) of peripheral nerves is increasingly utilized for nerve dysfunction, particularly in the presence of entrapment, with multiple positive meta-analyses for its application in carpal tunnel syndrome [6-8]. Ultrasound-guided cervical sympathetic chain hydrodissection with D5W has been reported to treat chronic upper trunk pain [9]. Additionally, ultrasound-guided hydrodissection of the cervical plexus with D5W has been used to treat patients with post-traumatic stress disorder [10]. Therapeutic hydrodissection of the vagus nerve with D5W without local anesthetic for chronic pain has not previously been reported. The carotid sheath, enclosing the common carotid artery, internal jugular vein, and the vagus nerve, provides a well-defined anatomical target for precise needle placement, and the method involves approaching the vagus nerve at C6-C7 level while hydrodissecting, separating it from the carotid artery, and surrounding it with D5W without local anesthetic. Ultrasound guidance provides the requisite accuracy and safety for the procedure, minimizing the risk of complications.

We present a unique symptom combination of chronic multisite pain, cardiac abnormalities, and other primary-organ-dysfunction symptoms in a middle-aged male patient. Ultrasound-guided hydrodissection with D5W without local anesthetic of the bilateral vagus nerves within the carotid sheath resulted in a remarkable response. This case report aims to highlight the potential effectiveness of ultrasound-guided vagus nerve hydrodissection using D5W in managing vagal-insufficiency-related recalcitrant chronic pain and organ dysfunction. The absence of local anesthetic solution in the injectate enables bilateral treatment of the vagus nerves without the concern of undesirable blocking effects on the bilateral vagus nerves and related nervous structures.

## Case presentation

The patient, a 49-year-old pastor, presented with a three-year history of persistent pain involving multiple sites, including the neck, bilateral shoulders, thoracic region, lower back, and bilateral knees. The right side was reported to be more severely affected than the left, likely due to the patient's right-hand dominance in daily activities. The pain was characterized as sore, dull, weak, and heavy, with a maximum pain rating of 9 on a 0-10 numerical pain rating scale. In addition to chronic pain, the patient experienced shortness of breath upon mild exertion, significantly impacting his ability to carry on longer conversations or deliver speeches. He sat intermittently in an attempt to reduce symptom severity. He also complained of limited appetite with early satiety, intermittent nausea, and regurgitation. 

The patient had a rapid resting heart rate of 100-120 beats per minute, which was not postural in nature. He also experienced occasional palpitations. The resting electrocardiogram (EKG) showed sinus tachycardia and premature ventricular complexes (PVCs). A 24-hour ambulatory EKG monitoring study confirmed the presence of 15% PVCs, including bigeminy and trigeminy patterns. During the PVCs, he occasionally needed to cough to ease the discomfort in his chest. Echocardiograms and computer tomography (CT) coronary angiograms performed over two consecutive years were both normal. Despite comprehensive evaluations by two cardiologists, including normal findings on CT coronary angiography for two consecutive years, no cardiac abnormalities were identified. He was put on a beta-blocker for symptom control, but the PVCs persisted, and rate change was minimal. Consultations with two respiratory physicians similarly yielded normal results for the respiratory system. Early satiety, intermittent nausea, and regurgitation complaints were not explained or improved by gastroenterology evaluation, dietary interventions by a dietitian, or proton pump inhibitors.

The patient had no significant past medical history or prior injury/surgery before the onset of his chronic pain. He had undergone a comprehensive evaluation by specialists across various medical disciplines, but the findings were either negative or inconclusive. Rheumatologists had diagnosed him with fibromyalgia and recommended physiotherapy and oral medication which ranged from maximal dose of paracetamol and tramadol, non-steroidal anti-inflammatory drugs (NSAIDs), muscle relaxants, benzodiazepam, gabapentin, and amitriptyline, which provided only minimal relief. Orthopedic surgeons ordered a dual-energy X-ray absorptiometry (DEXA) scan that was normal, and X-rays of his spine and knees revealed only mild, age-related degenerative changes. Apart from the trigger points, there were no other clear physical findings. He was then labeled as a "psychosomatic" patient and referred for psychiatric assessment, which did not reveal any significant abnormalities. a trial of antidepressant medication did not improve any symptoms.

The patient's Oswestry Disability Index (ODI) was 45%, consistent with substantial impairment of daily functioning and quality of life. Other intervention attempts had included 2.5 years of physiotherapy, ultrasound-guided trigger point injections, cervical and lumbar sympathetic blocks, and intravenous ketamine injections by other practitioners, without appreciable or sustainable benefit.

On physical examination during the patient's initial visit to our center, a neurological examination of the upper and lower limbs revealed no abnormalities. Musculoskeletal examination demonstrated normal range of motion in the neck, lower back, and bilateral upper and lower limbs. Palpation identified multiple trigger points in paraspinal muscles, particularly on the right side.

**Figure 1:**
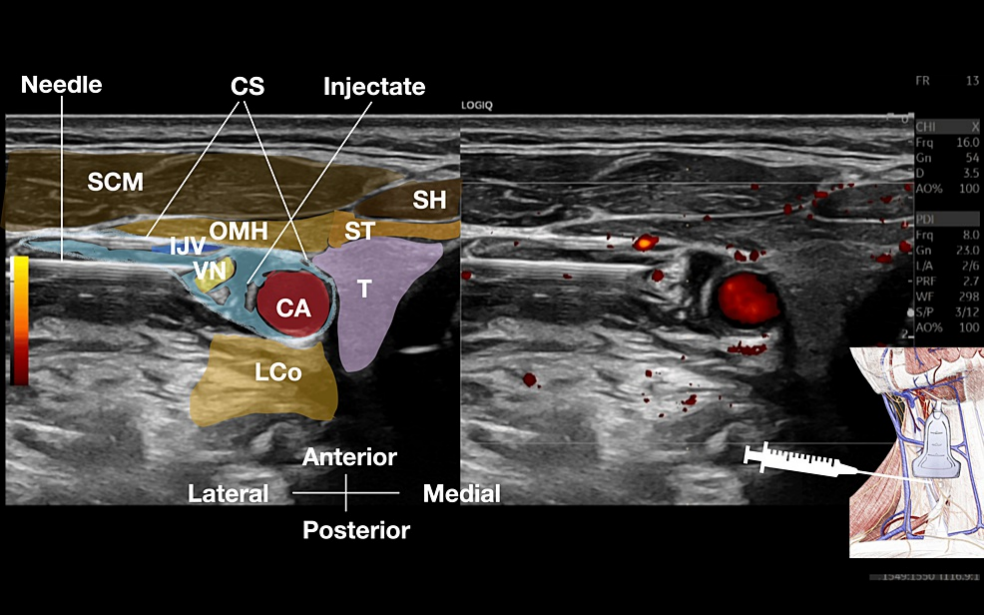
Ultrasound-guided hydrodissection of the vagus nerve within the carotid sheath using 5% dextrose as the injectate. CA, Carotid Artery; CS, Carotid Sheath; IJV, Internal Jugular Vein; LCo, Longus Coli; OMH, Omohyoid; SCM, Sternocleidomastoid; SH, Sternohyoid; ST, Sternothyroid, T, Thyroid, VN, Vagus Nerve

After obtaining informed consent, ultrasound-guided bilateral vagus nerve hydrodissection using D5W without local anesthetic was performed (Figure 1 and Video 1). The procedure was performed at time 0, one month, and two months. Maximum pain levels decreased from 8/10 pre-treatment to 3/10 immediately after treatment one, rose to 6/10 before treatment two, dropped to 2/10 immediately after treatment two, rose to 5/10 just before treatment three, and decreased to 2/10 immediately after treatment three. The patient felt the neck was lighter and the pain level gradually decreased while the D5W was slowly injected into the halo created around the vagus nerve. No complications of bradycardia, hypotension, swallowing, and voice dysfunction occurred during and post the vagus nerve hydrodissection.

**Video 1:** Ultrasound-guided hydrodissection of the vagus nerve in the carotid sheath using 5% dextrose without local anesthetic Detailed sonoanatomy of each step of the procedure is presented as labelled still images embedded within the video.

After the third treatment, the pain was maintained at 2-3/10 upon follow-up at three, six, and nine months following the last treatment. Physical examination revealed that the bilateral paraspinal muscle trigger points had largely subsided. The ODI score improved to 10% at three months, and the effects persisted at the six-month and nine-month follow-ups. Shortness of breath improved, enabling the patient to deliver speeches without interruption. Cardiac symptoms resolved, and there were no noticeable PVCs. Resting heart rate dropped to a mean of 70-80 beats per minute, and early satiety, nausea, and reflux complaints resolved without the need for medication. 

The technique

Patient Positioning

The patient is positioned supine with the head turned slightly away from the side being treated, and the head slightly hyperextended to better expose the anterior neck region, similar to positioning for ultrasound-guided cervical intervertebral disc injection [11,12]. 

Ultrasound and Transducer Placement

To optimize ergonomics, the ultrasound machine was positioned on the contralateral side of the neck being treated. A high-frequency linear ultrasound transducer, for example, GE ML4-20D (General Electric Company, Boston, Massachusetts, United States), was then placed transversely on the anterior neck, superior to the clavicle, at the level of C6-C7 vertebrae. This transducer placement allows for clear visualization of the carotid sheath and its internal structures.

Needle Guidance

Under continuous ultrasound guidance, a 25G or 27G 2-inch hypodermic needle is advanced using continuous hydrodissection from the point of skin entry both to, through, and within the carotid sheath, employing an in-plane technique [13]. The aim is to distribute the injectate around the vagus nerve, ensuring the fluid completely surrounds the vagus nerve. The hydraulic pressure of the D5W pushes away the soft tissues in front of the needle tip, preventing damage to the surrounding soft tissues. The volume effect of the injectate causes the internal jugular vein to collapse (Figure 1, Video 1) [14-16]. The procedure is then repeated on the opposite side. The cervical skin is usually thin and the adipose tissue is usually not as abundant as in other parts of the body, therefore the hydrodissection and needle advancement should be performed slowly. The whole procedure on one side from skin entry to completely hydrodissecting the vagus nerve from surrounding soft tissues inside the carotid sheath typically takes about six to nine minutes (Videos 2, 3). 

**Video 2:** A complete ultrasound-guided hydrodissection of the left vagus nerve in the carotid sheath using 5% dextrose without local anesthetic

**Video 3:** A complete ultrasound-guided hydrodissection of the right vagus nerve in the carotid sheath using 5% dextrose without local anesthetic

Injectates

After administering local skin anesthesia, a solution of 5% dextrose [17,18] without local anesthetic is the injectate used for both hydrodissection during needle advancement, and for completion of the hydrodissection process inside the carotid sheath. Typically a volume of 30-40 mL of D5W is required to hydrodissect from subcutaneous tissues into the carotid sheath and fully release the vagus nerve from the carotid artery. Empirical observations of bilateral vagus nerve hydrodissection using D5W under continuous vital sign monitoring in other patients have not revealed any tendency to bradycardia. Notably, perineural injection with dextrose for other nerves appears to restore normal baseline function rather than “stimulate” nerves [6,8]. 

Intra-and-Post-Procedure Monitoring

Throughout the vagus nerve hydrodissection procedure, the patient's status is closely monitored. This includes regular assessment of the patient's voice and swallowing function. The patient remains awake throughout the entire procedure, and the clinician performing the procedure communicates with the patient frequently, checking in between each syringe exchange. The patient is instructed to immediately report any discomfort they experience, such as pain or a sensation of electric shock. Vigilant monitoring of the patient's condition is crucial during this minimally invasive, ultrasound-guided vagus nerve hydrodissection. Blood pressure is measured before and after the procedure. Continuous vital signs, which include pulse and oxygen saturation are closely monitored during and immediately post the procedure for at least 15 minutes, for any immediate potential systemic responses to restoration of vagal tone, such as bradycardia or hypotension, or a temporary irritation of the vagus nerve from epineural contact by the needle, with possible vocal cord dysfunction. Even if the absence of bradycardic responses is formally confirmed by larger case collections or clinical trials, vital sign monitoring is recommended. 

Requirements of proper technique

Real-time adjustment of needle trajectory and clear visualization of the needle tip are needed at all times during needle advancement. Hydrodissection should be performed slowly from the point of skin entry to inside the carotid sheath, injecting D5W to push away soft tissues in front of the needle tip to create a halo before advancing the needle tip. This helps visualize and displace any potential obstacles and pushes away nerve branches too small to practically avoid. In order to safely hydrodissect the tract from skin entry to inside the carotid sheath and completely hydrodissect the vagus nerve from the surrounding tissues, typically 30-40 mL of D5W without local anesthetic is necessary for one side of this procedure. The procedure on one side, from skin entry to complete hydrodissection of the vagus nerve within the carotid sheath, typically takes six to nine minutes.

Forming a safe injection space around the vagus nerve by creating a fluid-filled cavity facilitates complete injection of the injectate, enabling a more even distribution of the injectate around the vagus nerve, which may improve the efficacy of the procedure. Closer monitoring of the vital signs before, during, and after the VN hydrodissection is crucial. Using continuous cardiac monitor throughout the entire procedure and for 15 minutes post procedure is necessary. 

## Discussion

Chronic pain is a complex and debilitating condition and often results from tissue damage with inflammation and pathological adaptation of the peripheral and central nervous system, including the autonomic system. In addition, insufficient vagal tone may result in the inability to mitigate the unfavorable effects of inflammatory cytokines, including the reduction of pain thresholds [2,3]. Either primary or secondary dysfunction of the vagus nerve may render conventional treatments for chronic pain less effective and associated with undesirable side effects. The vagus nerve is critical in regulating cardiac activity, respiratory function, gastrointestinal processes, immune, endocrine, and other autonomically mediated processes. 

Recently, VNS has emerged as a promising therapeutic approach for chronic pain management. Recent reviews concluded that VNS can regulate and normalize various bodily functions [19,20], and provide an analgesic effect [3]. Potential analgesic mechanisms of VNS are likely multiple, including modulation of descending pain pathways, anti-inflammatory effects, and reduction of central sensitization [2]. Clinical outcomes varied across studies, with a majority reporting a significant reduction in pain intensity, increased pain thresholds, and improved quality of life in patients receiving VNS therapy for chronic pain [3]. Adverse events associated with VNS were generally mild and well-tolerated. 

To date, to the best of our knowledge, no other studies have evaluated the specific effects of hydrodissection on the VN or the direct effects of having D5W surrounding the VN. However, restoration of function in multiple peripheral nerves as a result of hydrodissection has been demonstrated in randomized trials of the cubital tunnel and carpal tunnel syndrome, and corroborated by meta-analyses of carpal tunnel syndrome trials [6-8]. The therapeutic effects are attributed to both the release of nerves from fascial restrictions as seen in a volume effect study [21], and the direct therapeutic properties of the injectate, D5W [17,18], which has been the primary injectate reported in recent literature due to its availability, safety, and consistent clinical performance [6-8]. 

This is the first in the literature, as per our review, to report the use of ultrasound-guided vagus nerve hydrodissection to treat chronic, recalcitrant, multi-site pain, and autonomically-related-systemic symptoms. Similar to the effects demonstrated with the median nerve clinical trials, we postulate that the effects of hydrodissection may be related to both the mechanical separation of the vagus nerve from the surrounding soft tissues, as well as the pharmacological effects of the D5W solution when it completely surrounds the nerve.

The vagus nerve can be identified and released directly from the carotid artery with the utilization of consistent and continuous ultrasound visualization of the needle tip. Proper hydrodissection technique is crucial to mitigate the risks of hematoma from a carotid or internal jugular intimal penetration, or irritation of, or trauma to, the vagus nerve. Using D5W without local anesthetic as the injectate avoids the potential side effects associated with local anesthetics.

The substantial subsidence of multiple paraspinal trigger points observed in the current case suggests that vagus nerve hydrodissection may be an effective treatment for patients presenting with multiple recalcitrant trigger points. However, further large-scale studies are needed to thoroughly evaluate the effects of vagus nerve hydrodissection using 5% dextrose solution, without the addition of local anesthetics, for the management of myofascial pain.

## Conclusions

The successful treatment of this patient's chronic multisite pain and autonomic dysfunction symptoms through ultrasound-guided vagus nerve hydrodissection using 5% dextrose solution highlights the potential therapeutic value of this approach. Potential advantages of this technique include the ability to perform bilateral treatment, the mechanical component of hydrodissection which may facilitate durable effects, and the possibility of more rapid benefits compared to or in combination with other vagal nerve dysfunction treatments. This case report underscores the important role of vagal nerve dysfunction in contributing to chronic pain, suggesting that the term "sympathetically maintained pain" may overlook the significant impact of reduced vagal output.

The findings from this single case warrant further investigation through larger-scale, long-term studies to thoroughly evaluate the efficacy, safety, and durability of ultrasound-guided vagus nerve hydrodissection for managing chronic pain and associated autonomic disturbances. Rigorous research is needed to confirm the therapeutic potential of this novel intervention and elucidate the underlying mechanisms by which modulating vagus nerve function can provide relief for patients suffering from complex, treatment-refractory chronic pain conditions. Collaborative, multi-center trials are essential to generate high-quality evidence that can guide future clinical practice and improve outcomes for individuals burdened by chronic pain and autonomic dysregulation.

## References

[1] Gureje O, Von Korff M, Simon GE, Gater R (1998). Persistent pain and well-being: a World Health Organization study in primary care. JAMA.

[2] Olofsson PS, Rosas-Ballina M, Levine YA, Tracey KJ (2012). Rethinking inflammation: neural circuits in the regulation of immunity. Immunol Rev.

[3] Courties A, Berenbaum F, Sellam J (2021). Vagus nerve stimulation in musculoskeletal diseases. Joint Bone Spine.

[4] Hilz MJ (2022). Transcutaneous vagus nerve stimulation - a brief introduction and overview. Auton Neurosci.

[5] Goggins E, Mitani S, Tanaka S (2022). Clinical perspectives on vagus nerve stimulation: present and future. Clin Sci (Lond).

[6] Sveva V, Farì G, Fai A (2024). Safety and efficacy of ultrasound-guided perineural hydrodissection as a minimally invasive treatment in carpal tunnel syndrome: a systematic review. J Pers Med.

[7] Lam KH, Wu YT, Reeves KD, Galluccio F, Allam AE, Peng PW (2023). Ultrasound-guided interventions for carpal tunnel syndrome: a systematic review and meta-analyses. Diagnostics (Basel).

[8] Zhou T, Wu Z, Gou X, Xia H, Ding J, Ai S (2023). Local injection therapy for carpal tunnel syndrome: a network meta-analysis of randomized controlled trial. Front Pharmacol.

[9] Lam SK, Reeves KD, Cheng AL (2017). Transition from deep regional blocks toward deep nerve hydrodissection in the upper body and torso: method description and results from a retrospective chart review of the analgesic effect of 5% dextrose water as the primary hydrodissection injectate to enhance safety. Biomed Res Int.

[10] Reeves KD, Shaw J, McAdam R (2022). A novel somatic treatment for post-traumatic stress disorder: a case report of hydrodissection of the cervical plexus using 5% dextrose. Cureus.

[11] Lam KH, Hung CY, Wu TJ (2020). Ultrasound-guided cervical intradiscal injection with platelet-rich plasma with fluoroscopic validation for the treatment of cervical discogenic pain: a case presentation and technical illustration. J Pain Res.

[12] Lam KH, Hung CY, Wu TJ (2022). Novel ultrasound-guided cervical intervertebral disc injection of platelet-rich plasma for cervicodiscogenic pain: a case report and technical note. Healthcare (Basel).

[13] Narouze S, Peng PW (2010). Ultrasound-guided interventional procedures in pain medicine: a review of anatomy, sonoanatomy, and procedures. Part II: axial structures. Reg Anesth Pain Med.

[14] Lam KH, Hung CY, Chiang YP, Onishi K, Su DC, Clark TB, Reeves KD (2020). Ultrasound-guided nerve hydrodissection for pain management: rationale, methods, current literature, and theoretical mechanisms. J Pain Res.

[15] Lam KH, Lai WW, Ngai HY, Wu WK, Wu YT (2023). Comment on the safety of the ultrasound-guided hydrodissection technique for carpal tunnel syndrome. J Ultrasound.

[16] Lam KH, Lai WW, Ngai HY, Wu WK (2022). Practical considerations for ultrasound-guided hydrodissection in pronator teres syndrome. Pain Med.

[17] Cherng JH, Chang SJ, Tsai HD (2023). The potential of glucose treatment to reduce reactive oxygen species production and apoptosis of inflamed neural cells in vitro. Biomedicines.

[18] Wu YT, Chen YP, Lam KH, Reeves KD, Lin JA, Kuo CY (2022). Mechanism of glucose water as a neural injection: a perspective on neuroinflammation. Life (Basel).

[19] Shao P, Li H, Jiang J, Guan Y, Chen X, Wang Y (2023). Role of vagus nerve stimulation in the treatment of chronic pain. Neuroimmunomodulation.

[20] Wileczek A, Polewczyk A, Kluk M, Kutarski A, Stec S (2021). Ultrasound-guided imaging for vagus nerve stimulation to facilitate cardioneuroablation for the treatment of functional advanced atrioventricular block. Indian Pacing Electrophysiol J.

[21] Wu YT, Chen SR, Li TY, Ho TY, Shen YP, Tsai CK, Chen LC (2019). Nerve hydrodissection for carpal tunnel syndrome: a prospective, randomized, double-blind, controlled trial. Muscle Nerve.

